# Exercise and tissue-resident memory T cells: from circulating numbers to spatial immune remodeling

**DOI:** 10.3389/fimmu.2026.1822561

**Published:** 2026-05-07

**Authors:** Enli Xie, Yushan He, Tainan Cao, Changchun Li, Zhiming Wang

**Affiliations:** 1 Department of Strength and Conditioning, School of Sports Training, Nanjing Sports Institute, Nanjing, China; 2 School of Physical Education, Jingdezhen College, Jingdezhen, China

**Keywords:** adrenergic signaling, CD103, CD69, exercise immunology, lymphocyte homing, mucosal immunity, tissue-resident memory T cells, T_RM_

## Abstract

Tissue-resident memory T (T_RM_) cells have become a paradigm shift in the field of immunology and have changed our view of local immune surveillance at barrier surfaces. In contrast to circulating memory T cells, T_RM_ cells are fixed in non-lymphoid organs like lungs, intestine and skin to act as the first line of defense against reinfection and malignant conversion. The finding contradicts the conventional emphasis on exercise immunology on the number of circulating lymphocytes and requires a new conceptual framework of the so-called quantitative to spatial immune remodeling. This review summarizes the current developments in the T_RM_ cell biology and its relevance to exercise immunology and answer the main question of the review: will endurance training, like a vaccine, elevate the density of both T_RM_ cells in non-lymphoid tissues and their functional capacity? To begin with, we define the molecular basis of T_RM_ cells, their differentiation routes, storage processes (CD69, CD103), and tissue-specific diversity. Then, we explore the possible ways in which exercise might be able to change the establishment of T_RM_ cells and their functions, namely exercise related adrenergic signaling, thermoregulatory shifts, and hemodynamic forces regulating the T cell homing receptor and tissue resident program. The traditional finding of decreased upper respiratory tract infection risk in athletes is reevaluated based on the perspective of increased respiratory mucosal T_RM_ cell immunity. We then elaborate on the biphasic J-shaped relationship between exercise intensity and immunoprotection, and elucidate how optimal training levels are achieved at lower intensities whereas higher intensities can undermine the level of T_RM_ cell-mediated immunity. Lastly, we have identified key knowledge gaps and research directions that are needed in the future, namely, the mechanistic analysis of β2-adrenergic receptor signaling in T_RM_ cell biology, the creation of tissue-specific exercise prescription strategies and the translation of these findings into practice as a way to prevent infections and treat cancer immunotherapy. Incorporating basic immunology with exercise physiology, the review is intended to trigger a paradigm shift in exercise immunology shifting away the circulating numbers toward the spatially-resolved insight of how exercise alters the immune picture of the tissues.

## 1 Introduction

The field of exercise immunology has long been based on the quantification of circulating immune cells. In the last thirty years, it has been shown that acute exercise releases lymphocytes of the lymphoid reservoirs into the systemic blood circulation, whereas the chronic training can change the composition and functional properties of the circulating T cell subsets (1, 2). The concepts of the “open window” theory and the “J-shaped” curve model have offered useful paradigms in explaining the correlation between exercise dose and infection risk especially upper respiratory tract infections (URTIs) among athletes (3). Nevertheless, these models inherently limit themselves to the attention paid to circulating lymphocytes as a single compartment that is merely a small part of the overall body immune cell pool.

The last decade has seen an unprecedented change in the field of immunology that has essentially undermined the notion of circulating-centricity. It was found in 2009 by Gebhardt et al. that upon resolution of skin infection, a population of memory CD8^+^ T cells remains in the tissue and is able to give greater protection against re-infection than circulating memory cells (4). Such cells known as tissue-resident memory T (T_RM_) cells are immobile and remain lodged in non-lymphoid tissues such as skin, lungs, gut, reproductive tract, liver and kidneys (5–7). Later studies have shown that T_RM_ cells are the largest number of memory T cells in the body, and this number is about several times higher than that of their circulating counterparts (8).

T_RM_ cells discovery opens new vistas in the field of immune surveillance. T_RM_ cells are localized at the pathogen entry points where they are like local sentinels that can send an emergency alert signal to all other tissues in response to antigen re-encounter (9). They have a distinctive biology that relies on a central transcriptional program that is regulated by transcription factors like Hobit, Blimp-1, and Runx3 which reduce the expression of tissue-egress proteins (S1PR1, CCR7) and increase the expression of retention molecules like CD69 and CD103 (10, 11). Tissue microenvironment offers vital maintenance information such as TGF-beta in the induction of CD103 and IL-15 in the homeostatic proliferation (12, 13).

The new T_RM_ cell paradigm that is being born is putting some very important questions in the exercise immunology. In case T_RM_ cells are the major effector cells of local immune protection, can exercise training, especially endurance exercise, influence this process of T_RM_ cell establishment and maintenance? Is it possible that repeated exercise sessions could, via cumulative adrenergic signaling and hemodynamic alterations, train T cells to assume tissue-resident phenotypes? Even more provocatively, could exercise have functions similar to those of a vaccine, making protective T_RM_ cells more densely populated at susceptible areas including the respiratory mucosa?

A number of lines of evidence indicate that this hypothesis has mechanistic potential. Intense exercise promotes significant catecholamine secretion, especially epinephrine, which is able to affect β2-adrenergic receptors (β2-AR) on lymphocytes (14). β2-AR signaling has been found to control lymphocyte trafficking, adhesion molecule expression, and effector activity (15). In addition, effector memory T cells, which have similar phenotypic characteristics as T_RM_ cell precursors, express a high level of β2-AR, and are selectively mobilized during exercise (16). The rise in core body temperature and hemodynamic shear stress due to exercise can also play a role in regulation of T cell homing receptors expression and tissue extravasation (17). Continuous exercise could hypothetically offer regular signals that induce T_RM_ cell differentiation and survival in the peripheral tissues.

Clinical importance of this hypothesis is highlighted by the fact that it is documented that frequent moderate intensity exercises lower the risk of URTI by 20–30 percent and sedentary lifestyle and overtraining can raise vulnerability (18). In spite of the fact that this so-called J-curve correlation has been highly statistically verified, the cellular processes involved have not been fully elucidated. T_RM_ cells that line respiratory mucosa are ideally located to deliver such protection because they offer an immediate antiviral response at the port of entry (19). Insight into the mechanism underlying this long-standing observation could be gained by understanding how exercise influences T_RM_ cell immunity and this information can be used to provide evidence-based exercise advice on the health of your immune system.

This review will correlate the cell biology of T_RM_ with the immunology of exercise. Firstly, the molecular basis for the development, retention, and heterogeneity of T_RM_ cells will be discussed. Secondly, the role of exercise in the development and function of T_RM_ cells will be reviewed. Finally, the role of exercise in the development of URTIs will be readdressed in the context of T_RM_ cells. Gaps in the current knowledge will be identified to provide insight for the way forward in the field.

## 2 T_RM_ cell biology: molecular foundations and tissue heterogeneity

### 2.1 Discovery and definition of T_RM_ cells

The traditional categorization of memory T cells as either central memory (T_CM_) or effector memory (T_EM_) subsets using CCR7 and CD62L expression has offered a lasting model of T cell memory (20). Nevertheless, the dichotomy could not account for findings indicating that other memory T cells were present in peripheral tissues indefinitely without circulating back to the blood. This paradox was explained by the groundbreaking research of Gebhardt et al., in 2009, who showed that after herpes simplex virus infection, a group of CD8^+^ memory T cells stayed in the skin permanently, offering increased resistance to reinfection (4). Parabiosis experiments that followed, i.e. surgically connected mice sharing circulation, finally proved that these cells are indeed resident, unable to achieve equilibrium between partners (8, 21).

The new definition of T_RM_ cells involves three major characteristics, which include: (1) prolonged presence in non-lymphoid tissues with limited or no circulation in blood or lymph, although the degree of residency can vary by tissue—for instance, lung T_RM_ populations may decline over time; (2) prolonged existence after eliminating the antigens; (3) fast effector response to a repeat encounter with an antigen (22). They have been found in almost every non-lymphoid tissue, such as skin, lungs, intestine, reproductive tract, liver, kidneys, salivary glands, and brain (5–7, 23). T_RM_ cells contain both CD8^+^ and CD4^+^ subsets but CD8^+^ T_RM_ cells have been studied better because they have a strong cytotoxic effect (24).

### 2.2 Molecular hallmarks of T_RM_ cells

**Table 1** contains the summary of the main molecular markers of T_RM_ cells and their functions. The most widely used T_RM_ cell marker is CD69, a primary activation antigen that is constantly present on T_RM_ cells (25). CD69 causes retention in the tissue through its binding to and degrading sphingosine-1-phosphate receptor 1 (S1PR1) that can be utilized by lymphocytes to leave tissues in response to S1P gradients (26). CD69 also forms complexes with and blocks S1PR1 at the surface of the cell and also cannot escape the tissue (27). Although the activated circulating T cells upregulate CD69 temporarily, high-level expression is a hallmark of T_RM_ cells.

**Table 1:** Molecular markers of tissue-resident memory T cells.

Marker	Expression pattern	Primary function	Tissue distribution
CD69	Constitutive high expression	Binds S1PR1, prevents tissue egress	All T_RM_ cells
CD103 (αEβ7)	Epithelial T_RM_ cells	Binds E-cadherin, epithelial anchoring	Skin, lung, intestine, reproductive tract
CD49a (VLA-1)	Subset of T_RM_ cells	Binds collagen, interstitial retention	Skin dermis, lung interstitium
CD11a (LFA-1)	Broad expression	Adhesion to ICAM-1	Most T_RM_ cells
CCR4	Skin T_RM_ cells	Skin homing	Skin
CLA	Skin T_RM_ cells	E-selectin binding, skin homing	Skin
CCR9	Small intestine T_RM_ cells	Small intestine homing	Small intestine
α4β7	Gut T_RM_ cells	MAdCAM-1 binding, gut homing	Intestine
CXCR6	Lung T_RM_ cells	CXCL16 binding, lung retention	Lung
CD44	High expression	Hyaluronan binding, adhesion	Most T_RM_ cells

CD103 is a marker of T_RM_ cells in epithelial tissues such as skin, lung, and intestine (28). CD103 has been found to interact with E-cadherin present on epithelial cells, thereby tethering T_RM_ cells to the epithelial layer (29). The expression is activated by TGF-β signaling that is abundant in the microenvironment of the epithelial tissues (12). It is interesting that CD103 expression is tissue-specific; T_RM_ cells in liver and kidney do not express CD103 as they are devoid of any epithelial structure (30).

Other adhesive molecules are involved in the maintenance of T_RM_ cells in defined tissue niches. CD49a (integrin alpha 1 beta 1) is also a collagen binding molecule that facilitates retention in both dermis and lung interstitium (31). CD11a (LFA-1) is responsible to interact with ICAM-1 on endothelium and in tissues (32). Tissue-specific homing receptors such as CCR4 and CLA in skin, CCR9 and α4β7 in intestine, and CXCR6 in lung direct T_RM_ cell precursors to their respective sites of destination in the tissues (33–35).

### 2.3 Differentiation and transcriptional regulation

**Figure 1:**
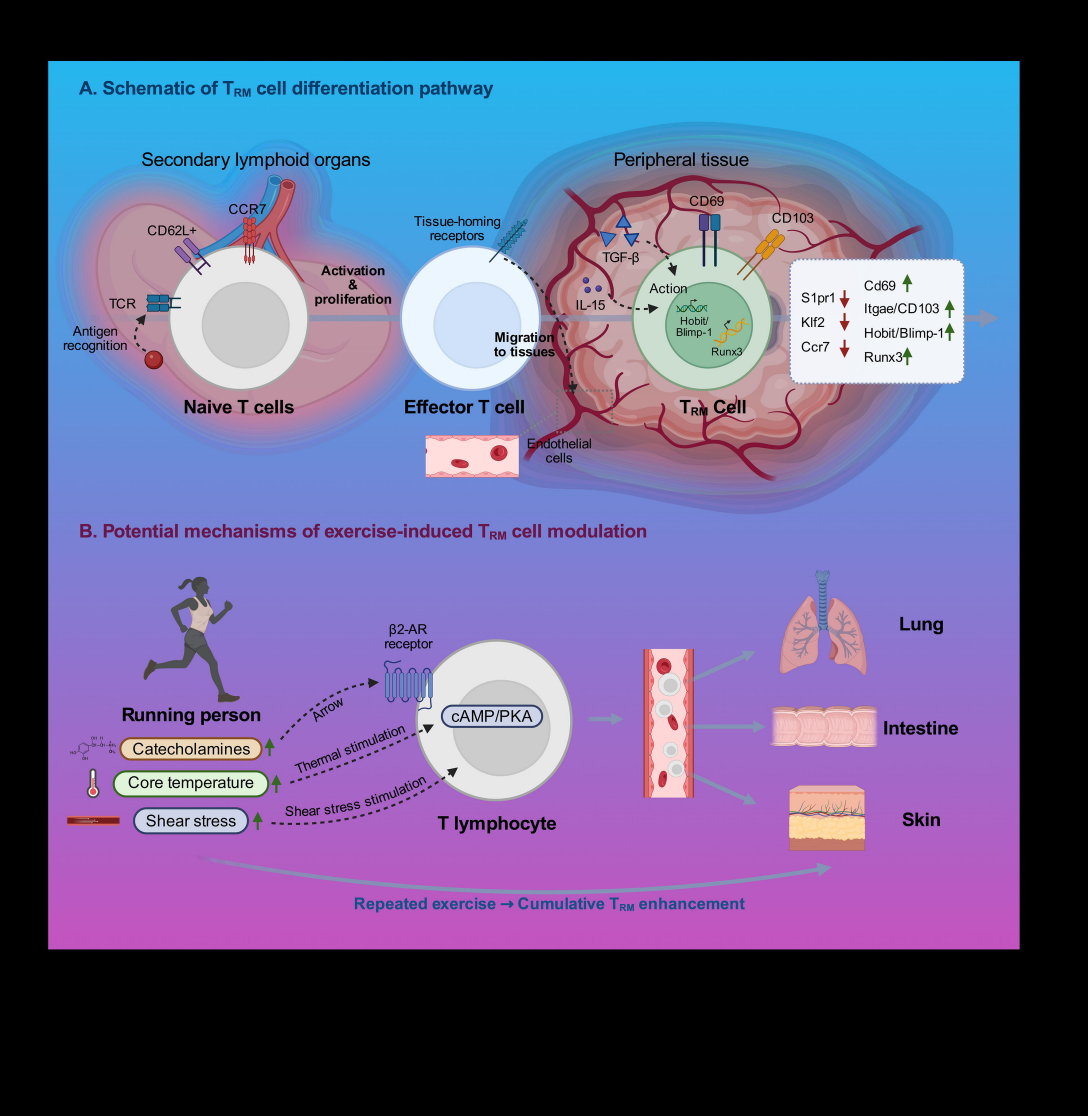
T_RM_ cell differentiation and exercise-induced modulation. **(A)** Schematic of T_RM_ cell differentiation pathway: Naive T cells stimulated in secondary lymphoid organs develop into effector T cells which go to peripheral tissues. In the tissue microenvironment, cues such as TGF-1, IL-15, and antigen persistence can drive downregulation of circulating-related genes (S1pr1, Klf2, Ccr7) and upregulation of residency-related genes (Cd69, Itgae, and transcription factors Hobit/Blimp-1, Runx3), and this leads to fully developed T_RM_ cells. **(B)** Possible ways of regulating T_RM_ cells through exercise. Exercise-induced catecholamines act on T cells at the β2-AR, inducing tissue homing. The higher core temperatures and hemodynamic shear forces might have an effect on the expression of adhesion molecules and transendothelial migration. Cumulative enhancement of T_RM_ cell establishment in peripheral tissues may occur due to repeated exercise sessions.

T_RM_ cell differentiation is a multi-stage event involving the integration of information on antigen recognition, inflammation and microenvironment of the tissue (**Figure 1A**). After being activated within secondary lymphoid organs, naive T cells become effector T cells that relocate to peripheral tissues (36). A fraction of effector cells in the tissue microenvironment has been shown to start a transcriptional program that down-regulates circulating associated genes (S1pr1, Klf2, Ccr7) and up-regulates residency associated genes (37).

The network of transcription factors that regulates T_RM_ cell differentiation has been well characterized. Hobit (homologue of Blimp-1 in T cells) and Blimp-1 (B lymphocyte-induced maturation protein-1) are known as master regulators of tissue residency, which directly inhibit S1pr1 and Klf2 expression (11). Mice that do not have both Hobit and Blimp-1 in T cells cannot produce T_RM_ cells in a variety of tissues. Runx3 (Runt-related transcription factor 3) supports the process of CD8^+^ T_RM_ cell differentiation through the induction of CD103 expression and its consolidation into the resident program (38). The role of the Notch signaling pathway is in T_RM_ cell survival and persistence, especially in skin and gut (39).

Signals of cytokines in the tissue microenvironment play a critical role in establishing and sustaining T_RM_ cells. Epithelial cells and stromal cells produce TGF-β which up-regulates the expression of CD103 via a Smad-dependent pathway and down-regulates T-bet, a transcription factor that drives circulating phenotypes (12, 40). IL-15 is providing crucial survival signals, supporting homeostatic proliferation by activating STAT5 (41). In some tissues, IL-7 is involved in the maintenance of T_RM_ cells, but its contribution is not as significant as IL-15 (42).

### 2.4 Tissue-specific heterogeneity

The T_RM_ cells are characterized by a stunning functionality and phenotype that is adjusted to the residing tissues, which is the result of the individual needs and signals of each microenvironment (**Table 2**). Lung T_RM_ cells are located at the border between the external worlds and need to balance between high sensitivity and the ability to tolerate inhaled antigens (43). They have CXCR6, which binds to CXCL16 in airway epithelial cells, and facilitates their retention in the lung parenchyma (34). After influenza infection, lung T_RM_ cells offer heterosubtypic immunity by identifying common viral epitopes and limiting the severity of illness when confronted with drift strains (44).

**Table 2:** Tissue-specific features of T_RM_ cells.

Tissue	Distinguishing markers	Key maintenance signals	Primary functions	Pathological associations
Lung	CD69, CD103, CXCR6	TGF-β, IL-15	Antiviral defense (influenza, RSV, SARS-CoV-2)	COPD, asthma
Small intestine	CD69, CD103, CCR9, α4β7	IL-7, TGF-β, dietary antigens	Defense against enteric pathogens	Inflammatory bowel disease
Skin (epidermis)	CD69, CD103, CD49a	TGF-β, IL-15	Antiviral defense (HSV, VV)	Psoriasis, vitiligo, FDE
Skin (dermis)	CD69, CD4^+^ T_RM_ predominant	IL-7, IL-15	Helper functions, immune regulation	Cutaneous T cell lymphoma
Liver	CD69, CXCR6	IL-15	Defense against blood-borne pathogens	Chronic hepatitis
Female reproductive tract	CD69, CD103	TGF-β, IL-15	Defense against STIs (HSV-2, HIV)	Viral persistence

The intestinal T_RM_ cells are found in two separate compartments, the intraepithelial lymphocytes (IELs), which express CD103, and the lamina propria lymphocytes (LPLs), of a more varied phenotype (45). The intestinal microenvironment presents special challenges such as contact with commensal microbiota and dietary antigens. T_RM_ cells of the intestine have CCR9 and alpha4beta7 which direct them to the small intestine upon differentiation (33). They rely on IL-7 that is made by intestinal epithelial cells and stromal cells (46).

The T_RM_ population that was initially discovered, skin T_RM_ cells, has been well studied in murine models and in human skin (4, 47). Epidermal CD8^+^ T_RM_ cells can be recognized by a high expression of CD103 and CD49a, which anchor these cells in the keratinocyte layer and dermal T_RM_ cells are more heterogeneous phenotypically (31). Skin T_RM_ cells are resistant to cutaneous pathogen, such as herpes simplex virus and vaccinia virus (4, 48). They are also involved in recurring inflammatory skin disorders, such as psoriasis, vitiligo and fixed drug eruptions (49, 50).

The liver T_RM_ cells, located in the sinusoids, have different characteristics that are indicative of the special liver microenvironment (30). These cells express CXCR6 and CD69, but are usually not CD103, as expected in a non-epithelial structure. Liver T_RM_ cells offer immunity to blood-borne viruses such as Plasmodium and hepatotropic viruses (51).

### 2.5 Effector functions

The effector mechanisms that T_RM_ cells use to provide protection. When encountering an antigen again, T_RM_ cells respond quickly by generating inflammatory cytokines such as IFN-γ, TNF-α and IL-2 which triggers the local immune response and attracts circulating cells (9). The secretion of chemokine by epithelial cells and endothelial cells induced by IFN-γ production by T_RM_ cells causes a tissue-wide alarm response that increases the effectiveness of this response.

T_RM_ cells with CD8^+^ phenotype have a high cytotoxic potential and reactivate when stimulated and express granzyme B and perforins (52). They are able to destroy infected tissue parenchyma cells directly and suppress the dissemination of the pathogen prior to the initiation of circulating responses. Most significantly, T_RM_ cells do not need to undergo terminal differentiation in order to maintain their cytotoxic capability and consequently they remain present even after the pathogen has been cleared and can engage with new challenges (53).

CD4 ^+^ T_RM_ cells give helper roles, which assist in maintaining CD8 ^+^ T_RM_ cells and B cell responses (54). They will secrete different kinds of cytokines depending on where they reside in the body: TH1-like T_RM_ cells will secrete IFN-γ upon viral and intracellular bacterial infection; TH2-like T_RM_ cells will secrete IL-4, IL-5, and IL-13 in response to infection by helminthes or allergens; TH17-like TRM cells will secrete IL-17A and IL-22, which is essential in warding off extracellular bacteria and fungi (55, 56). New research has found that some CD4^+^ T_RM_ cells have the properties of T follicular helpers and help the local antibody response in mucosal tissues (57).

One singular characteristic of T_RM_ cells is their ability to sense and alarm when not being stimulated by cognate antigen. T_RM_ cells can sense inflammatory signals through cytokine receptors and pattern recognition receptors, and rapidly produce IFN-γ which stimulates the local innate and adaptive immune reactions (9). The sensing independent of the antigens will provide the fastest first line of defense against infections prior to the recognition of specific antigens.

### 2.6 T_RM_ cells in disease

T_RM_ cells have two functions: they are helpful in maintaining health but also cause autoimmune and inflammatory diseases by serving as an important protective mechanism against infection and cancers (**Figure 1B**).

Infectious diseases: T_RM_ cells are vital in the defense against a wide range of pathogens. Both the respiratory T_RM_ cells and circulating memory cells are more effective at protecting against influenza, respiratory syncytial virus, and SARS-CoV-2, but respiratory T_RM_ cells are more effective (19, 58). Intestinal T_RM_ cells protect against intestinal pathogens such as Listeria monocytogenes and Yersinia pseudotuberculosis (59). Skin T_RM_ cells offer long-lasting protection against HSV-1, HSV-2, and vaccinia virus (4, 48). Tract T_RM_ cells of female reproduction are also necessary to protect against sexually transmitted infections, such as HSV-2 and HIV (60).

Tumor-infiltrating: lymphocytes with T_RM_-like phenotypes have been correlated with better prognosis in a variety of cancers, such as melanoma, lung cancer, colorectal cancer, and breast cancer (61–63). In tumors, CD8^+^ T_RM_ cells have been shown to have higher effector functions and are also linked to responding to immune checkpoint blockade (64). They might be valuable targets in cancer immunotherapy because their tissue-resident phenotype would allow them to monitor the tumor over extended periods without becoming exhausted.

Autoimmune and inflammatory diseases: Persistent T_RM_ cells cause recurrent inflammation in a number of autoimmune diseases. Psoriasis is characterized by CD8^+^ T_RM_ cells that make IL-17 and are located at the site of previous psoriasis lesions and produce relapse when triggered (49). CD8^+^ T_RM_ cells that produce IFN-γ in the condition of vitiligo contribute to the gradual death of melanocytes (65). In fixed drug eruption, drug-specific CD8^+^ T_RM_ cells remain in lesions for decades and will rapidly recur on drug re-exposure (50). Inflammatory bowel disease has pathogenic T_RM_ cells that sustain mucosal inflammation (66).

## 3 Exercise immunology: classical concepts and emerging paradigms

### 3.1 The circulating lymphocyte paradigm

The study of exercise immunology has traditionally been based on circulating lymphocytes because of their easy availability and well-known functions as systemic immunity. An abrupt, massive redistribution of leukocytes is induced by acute exercise, and lymphocyte numbers increase up to 2–3 folds within a few minutes after exercise (2). This mobilization especially targets NK cells, CD8^+^ T cells, and γδ T cells that express large amount of β2-AR and respond strongly to catecholamine signaling (16, 67). Intensity and duration of mobilization are proportional to intensity and duration of exercise, which indicates that the proportionality of sympathetic activation is also proportional to intensity and duration of exercise.

After the end of exercise, there is a rapid decrease of lymphocytes with frequent counts that are lower than the pre-exercise values in recovery (68). This so-called lymphopenia indicates that cells have been redistributed between blood and tissues, not because of cell death, and usually disappears within several hours. Biphasic response-mobilization and then redistribution has been understood as a stress reaction which can transiently undermine immune surveillance which forms the essence of the open window hypothesis (69).

Long-term exercise training modifies the composition of circulating T cell compartments. The proportion of naive T cells is higher and senescent/differentiated T cells are lower among endurance athletes than sedentary people indicating that regular exercise prevents immunosenescence (70, 71). Such effects are believed to be due to decreased systemic inflammation, increased thymic output, and better homeostatic regulation (72).

### 3.2 The J-curve and upper respiratory tract infection risk

The association between exercise volume and URTI risk is related to a J-shaped curve: sedentary people are at a moderate risk of UTRI, but regular moderate exercise decreases the risk by 20–30 percent, and excessive training volumes (e.g., elite athletes during strenuous training) elevate the risk (18). It has been confirmed in many epidemiological investigations and regarded as one of the strongest results in the field of exercise immunology (3).

Beneficial aspects of moderate exercise are widely known. A systematic review of randomized controlled trial on the effect of regular moderate intensity exercises demonstrated a significant decrease of URTI occurrence (about 31%) in patients who performed the exercises in relation to those who were physically inactive (73). Adding one thousand extra steps to the daily routine have been linked to four fewer days of URTI symptoms every year (74). The results have clinical importance and imply less absenteeism and enhanced quality of life.

The causes of the J-curve have been researched a lot but not completely known yet. Light exercise can promote immune surveillance through mobilization of the effector lymphocytes, enhancement of the neutrophil phagocytosis and increase in the levels of salivary IgA secretion (75). The anti-inflammatory actions, such as decreased basal inflammation and increased IL-10 production, might be involved in the better regulation of the immune system (76). Conversely, vigorous activity causes the release of stress hormones, specifically cortisol, which inhibits lymphocyte activity and causes a transitory immunodeficiency (77).

### 3.3 Limitations of the circulating paradigm

Although this paradigm has explanatory strength, the circulating lymphocyte paradigm has underlying weaknesses to it. Most of the body lymphocytes are located in tissues, but not in blood - estimates suggest that circulating lymphocytes are fewer than 5 percent of the total lymphocytes (78). Especially, T_RM_ cells are fixed in tissues and never found in blood, hence they cannot be detected by conventional immune surveillance.

The association between the circulating and the tissue-resident compartment is not well-known. The variation in the number of circulating lymphocytes can be due to the redistribution between blood and tissues instead of the change in overall body immune status. As an illustration, the post-exercise lymphopenia might indicate increased lymphocyte emigration to tissues (which would be advantageous to immune surveillance) instead of immune suppression (79).

Furthermore, the J-curve relationship has mostly been explained using the concept of systemic immune suppression, although another explanation can be made: it could be that moderate exercise promotes the development of T_RM_ cells in the mucosal tissues, which offer better local protection, whereas overtraining may destroy the T_RM_ cells or their function. The hypothesis shifts the focus of the question on circulating number to tissue residency implying that spatial allocation of immune memory can be equally significant as its amount.

## 4 Exercise modulation of T_RM_ cells: potential mechanisms

### 4.1 Adrenergic signaling and β2-AR

The β2-adrenergic receptor (β2-AR) is the main intermediary in the mobilization of lymphocytes due to exercise and can also influence the development of T_RM_ cells (14). The β2-AR is a G protein-coupled receptor that is found on lymphocytes, and most highly expressed on effector memory and NK cells (16). Epinephrine, which is released by the adrenal medulla during exercise, is engaged and causes cAMP-PKA signaling, which leads to reorganization of the cytoskeleton and changes in expression of adhesion molecules.

The signal of the β2-AR stimulates the separation of the lymphocytes in the stroma niche in secondary lymphoid organs and allows these cells to migrate into the bloodstream (80). It is achieved through the process of the down-regulating of adhesive ligands such as L-selectins (CD62L) and CXCR4, which bind lymphocytes to the lymphoid tissue (81). Simultaneously, it was suggested that β2-AR signaling might also increase expression of the homing receptors to the tissue thus shifting the lymphocytes back into the periphery (82).

The importance of β2-AR signaling in T_RM_ cell biology is also backed up by a number of facts. To begin with, T_RM_ cell precursors (effector memory T cells) have large amounts of β2-AR and are selectively recruited during exercise (16). Secondly, β2-AR stimulation may regulate T-cell differentiation, and prolonged stimulation leads to the development of memory phenotypes (83). Thirdly, adrenergic signaling is capable of modifying T cell metabolism, which can impact long term survival and functionality (84).

The recent work involving selective beta-blockers has given mechanistic clues. Bisoprolol, a β1-selective blocker, was found to increase the mobilization of effector lymphocytes during exercise, indicating that the blockage of β1-AR promotes an increased availability of epinephrine to β2-AR (15). Nadolol, an unselective β-blocking agent, prevented mobilization, which supports β2-AR dependency. Such results prove the fact that the effect of adrenergic signaling could be controlled by pharmacology in order to boost effector lymphocyte mobilization, hence, having the opportunity to influence the development of T_RM_ cells.

The combined effect of repetitive workouts on T_RM_ cell development via the signal of β2-AR is not yet known. Prolonged exercise training increases the β2-AR expression in lymphocytes and makes them more susceptible to catecholamine (85). Such an adaptation might amplify the action of every exercise session thus causing increased efficiency of T-cell circulation into peripheral tissue followed by the differentiation of T_RM_ cell. It is important to note that while β2-AR mobilization is well established in human exercise studies, the extrapolation from mobilization to durable T_RM_ establishment remains mechanistic speculation at this stage.

### 4.2 Hemodynamic forces and shear stress

Exercise has a dramatic effect on cardiac output and blood flow rate, which causes a shear stress on endothelial cells and circulating leukocytes (86). Shear stress induces mechanosensitive ion channels and signaling pathways in endothelial cells leading to increasing expression of adhesion molecules such as ICAM-1 and VCAM-1 that facilitate lymphocyte adhesion and transmigration (87).

The mechanosensitive receptors, such as Piezo1, are expressed in lymphocytes and they react to shear stress by activating the calcium signal and rearrangement of the cytoskeleton (88). The mechanical forces produced during the exercise might also affect the activation state of lymphocytes, the expression of adhesion molecules, and their efficiency in transmigration. They might be especially important when it comes to lymphocyte entrance to high-flow vascular networks, such as the pulmonary circulation.

Exercise increases the cardiac output which in turn improves lymphocyte distribution to the peripheral tissues. The lung experiences very high lymphocyte flux because it is exposed to the whole cardiac output (89). This improved delivery may raise the rate at which circulating T cell precursors come into contact with their target tissues and start programs of tissue residency.

### 4.3 Thermal effects

Physical activity raises the core body temperature by 1–2 degrees Celsius with even higher increases in active muscles and local tissues (90). A mild hyperthermia affects various factors of immune function such as proliferation of lymphocytes and synthesis of cytokines and their migration (91).

The heat stress causes the expression of heat shock proteins (HSP) that are the molecular chaperones and regulate the immune response (92). The HSP70 has also been associated with T cell activation and survival. Lymphocyte survival in peripheral tissues may be enhanced by exercise-induced HSP expression thus helping to create conditions conducive to T_RM_ cell development.

In addition to heat shock proteins, T cells also express a heat-sensitive ion channel—transient receptor potential vanilloid 1 (TRPV1)—which acts as a molecular thermometer capable of detecting fluctuations in physiological temperature (93). Mild hyperthermia (within the range of exercise-induced core body temperature elevation, i.e., 38–40 °C) activates TRPV1, thereby triggering calcium influx and downstream signaling cascades, which in turn regulate T-cell activation, cytokine production, and cytoskeletal remodeling (94, 95). Recent evidence suggests that TRPV1 signaling enhances T-cell motility and promotes the expression of adhesion molecules such as CD11a, which may facilitate the transendothelial migration and tissue entry required for T_RM_ cell establishment (96). Thus, exercise-induced heat stress promotes T_RM_ cell differentiation not only through heat shock proteins but also via TRPV1-dependent pathways that optimize T-cell migration and tissue retention (94).

Elevation in temperature can also directly affect the trafficking of lymphocytes. It has been shown by *in vitro* experiments that slight hyperthermia improves T cell chemotaxis and transendothelial migration (97). The changes can be due to greater fluidity of cell membranes, more effective receptor signaling, or different cytoskeletal dynamics.

### 4.4 Metabolic regulation

Exercising causes significant alterations in metabolism such as greater glucose absorption and fats burning processes and production of mitochondria (98). The metabolic changes also go to immune cells that change their metabolic activity to suit the requirements of activation and effectors processes (99).

The T_RM_ cells have distinctive metabolic characteristics that allow them to persist over time. They increase the expression of fatty acid-binding proteins (FABP4 and FABP5) which allow the uptake and oxidation of exogenous fatty acids to generate energy (100). Through this metabolic adaptation, T_RM_ cells can survive in tissues where there is little or no available glucose and depend on the local lipid pool instead.

Exercise training can improve whole-body fatty acid oxidation and exercise training might have an impact on lipid availability of peripheral tissues (101). Steady exercise might also help in T_RM_ cell metabolism through elevation of local fatty acids levels and induction of FABP expression. In contrast, the metabolic stress elicited by exercise may enhance the fitness of mitochondria and clearance of autophagy which is needed to ensure the survival of lymphocytes over long periods (102).

### 4.5 Cytokine and myokine signaling

The contraction of skeletal muscles causes release of myokines that are cytokines and peptides that have endocrine and paracrine actions (103). The most researched myokine being IL-6, which is released with exercise and pleiotropically acts on immune cells (104). IL-6 supports T cell survival and effector function and possibly T cell differentiation (105).

IL-7 and IL-15, which are essential to maintain T_RM_ cells, are generated by various tissues such as muscles (106). Exercise training enhances muscle IL-15 expression, which might increase systemic IL-15 levels (107). The effect of exercise on the production of IL-7 in non-hematopoietic cells has also been studied but it cannot be directly supported by evidence.

IL-33 is an alarmin that is secreted during tissue damage and supports the maintenance of T_RM_ cells in barrier tissues (108). Tissue remodeling and mechanical stress caused by exercise may increase IL-33 accessibility, which might favor the survival of T_RM_ cells. Nevertheless, the hypothesis needs to be tested experimentally.

### 4.6 Epigenetic programming

New findings indicate that exercise brings about epigenetic changes in the immune cells such as DNA methylation and histones modification (109). It is believed that these epigenetic alterations affect how T cells differentiate and form memories of infections and this can affect the development of T_RM_ cell.

The idea of trained immunity is the improved innate immune responses after the first stimulus due to reprogramming of metabolism and epigenetics (110). Although originally described in innate immune cells, such mechanisms might be also used in adaptive lymphocytes. The epigenetic changes of exercise might be able to be used to train T cells to become more responsive, or drive memory differentiation.

The periodic signals provided by repeated exercise sessions may support epigenetic plans that promote tissue residency. Long-term exercise training of mice modifies the DNA methylation profile of T cells which also affects the expression of genes involved in migration and effector function (111). It is still unknown whether these alterations affect the establishment of T_RM_ cells.

## 5 T_RM_ cells and the J-curve: a reinterpretation

### 5.1 The classical observation

The J-shaped association between exercise amount and URTI susceptibility is perhaps the strongest finding in exercise immunology (18). The curve may be divided into three areas: (1) sedentary people who have a low to moderate risk of URTI; (2) those who exercise moderately with a 20–30 percent reduction in risk; (3) those who exercise with high volumes of activity with an increased risk especially when they are in peak physical condition or competitive situations (3).

The pathophysiological principles of the J-curve have been conventionally formulated in terms of systemic immunity: moderate exercises improve immune surveillance, whereas intense exercises cause stress-induced immunosuppression (77). Although these explanations are valid, they do not explain the tissue specificity of respiratory infection that is localized at mucosal surfaces and is mainly regulated by local immune actions.

### 5.2 Respiratory T_RM_ cells as mediators of protection

T_RM_ cells of the respiratory mucosa are optimally placed to defend against URTI. After influenza infection, T_RM_ cells of the lungs offer better protection than circulating memory, quickly inhibit viral replication and decrease the severity of the disease (44, 112). The T_RM_ cells that respond to the conserved viral epitopes give the heterosubtypic security against drifted strains (44).

Infection with SARS-CoV-2 causes long-lasting T_RM_ cell responses in the respiratory tract that are associated with immunity to reintroduction (58). Intranasal immunization that is more effective in producing respiratory T_RM_ cells than parenteral immunization, is more effective in preventing respiratory viral infection in animal models (113).

The results confirm respiratory T_RM_ cells as important mediators of mucosal immunity. The J-curve might then be redefined as indicating a shift in the density or functionality of respiratory T_RM_ cells with regard to exercise doses. T_RM_ cell establishment can be improved by moderate exercise in the respiratory mucosa whereas excessive training can adversely affect their maintenance or efficacy.

### 5.3 Exercise dose and respiratory T_RM_ cells

The relationship between exercise dose and respiratory T_RM_ cells can be hypothesized as follows:

**Sedentary state:** The sedentary state refers to a condition under which homeostatic markers like IL-15 and TGF-β are released to preserve baseline T_RM_ cell densities in the respiratory system due to the actions of the tissue microenvironment. Nonetheless, in the absence of intermittent signals that drive T cell trafficking and retention, the number of T_RM_ cells could be inadequate and hence there will be gaps in the local immune network.

Moderate exercise: Every exercise has periods of catecholamine release, hemodynamic shear stress and thermogenic signals that cause T cells to migrate into peripheral tissues such as the lung. Cumulative effect of repeated moderate exercise sessions positively affects T_RM_ cell formation with subsequent elevation of T_RM_ cell number in the respiratory mucosa. T_RM_ cell survival and function might also be supported by increased metabolic fitness and mitochondrial activity.

High-volume exercise: Excessive training is high volume physical activity. The overtraining can overwhelm the homeostatic abilities of T_RM_ cells. Increased levels of cortisol caused by stress might inhibit T cell survival and activity as an effector (77). Overtraining-associated systemic inflammation can lead to unfavorable micro conditions that disrupt the T_RM_ cell survival (114). Poor nutrition and metabolism can drain the substrates that are needed to support T_RM_ cell survival such as fatty acids to generate energy.

According to this model, respiratory T_RM_ cell density is expected to be negatively correlated with URTI risk at different exercise doses. To test this prediction, it is necessary to create ways of non-invasive assessment of human respiratory T_RM_ cells- a challenging task technically but a vital future perspective.

### 5.4 T_RM_ cells and exercise-induced asthma protection

Physical training alleviates the symptoms of asthma and enhances the quality of life of people with asthma (115). The pathophysiology of this advantage is not fully elucidated although it might be associated with the T_RM_ cell-mediated control of airway inflammation.

The TH2-type immune response plays a key role in allergic asthma, with IL-4, IL-5 and IL-13 driving the inflammatory process of eosinophils and airway hyperresponsiveness (116). TH2-biased CD4^+^ T_RM_ cells remain in the airways of patients with asthma and are considered as part of the disease chronicity (117). Exercise training may be able to alter the polarization of T_RM_ cells toward a direction where pathogenic TH2 responses are balanced out by protective TH1 or regulatory phenotypes.

Also, exercise-induced elevations in regulatory T cell counts or activity can reduce T_RM_ cell-driven inflammation (118). Treg cells have been shown to control T_RM_ cell responses in various settings such as skin and gut and possibly regulate pathogenic T_RM_ cells in the airways.

## 6 Translational implications and future directions

### 6.1 Exercise prescription for enhanced T_RM_ immunity

In case exercise training is able to improve the cell-mediated immunity of T_RM_ cells, exercise prescription must be optimized so as to achieve this goal. The main parameters that need to be investigated include:

Intensity: The J-curve postulates a specific intensity range in which T_RM_ cell enhancement will be achieved, and moderate intensity (50-70% VO2max) is the most effective. The intensity of high-intensity exercise (>80% VO2max) might be higher than optimal and negatively affect the maintenance of T_RM_ cells. The hypothesis can be tested by animal studies that may compare continuous moderate training with high-intensity interval training.

Frequency: There is no information on the cumulative impact of repeated exercise sessions on T_RM_ cell establishment. Daily exercise might be more efficient in keeping high T cell traffic in tissues than alternate-day exercises. Nevertheless, lack of sufficient recovery between the sessions may negatively affect maintenance of T_RM_ cells.

Time: Shorter exercise of 30–60 minutes has been shown to generate strong lymphocyte mobilization (2). It remains to be seen whether more extended periods offer extra value or added danger. The best time can vary depending on the need to create new T_RM_ cells or the requirement to preserve the current populations.

The relationship between time and infection/vaccination: Pre-vaccination or simultaneous with vaccination exercise may increase the production of T_RM_ cells through increasing the migration of T cells into tissues and influencing the inflammatory microenvironment (119). The timing of seasonal exercise interventions might be maximized to overlap with times when the threat of infection is higher (e.g., in winter).

### 6.2 T_RM_ cells as biomarkers of immune health

The present day immune monitoring in athletes depends greatly on circulating leukocytes counts, which do not give much insight into the tissue-resident immunity. Integration of T_RM_ cell-related biomarkers may be helpful in evaluating both the condition of the immune system and the likelihood of infection.

The direct sampling of respiratory T_RM_ cells has traditionally involved invasive interventions, but recent human studies demonstrate that nasal and nasopharyngeal swabs can recover local resident-memory populations in the upper airway (120). These non-invasive sampling techniques provide a feasible approach to assess T_RM_ like populations associated with respiratory protection, offering translational potential for immune monitoring in exercise settings. Mucosal immune status, which can be affected by T_RM_ cells, might be reflected by salivary IgA levels, which are predictive of URTI risk among athletes (75).

The circulating T cell subsets acting as T_RM_ cell precursors might offer indirect data. Memory effector T cells with the surface markers that are specific to the tissue (e.g., CD103, CCR9, CXCR6) can be considered as a new wave of tissue immigrants or cells with a future in tissue colonization (121). The frequency of these populations may be in relation to T_RM_ cell density in the respective tissues.

### 6.3 Exercise and vaccination synergy

Effective vaccines capable of inducing T_RM_ cell responses are better protectors against mucosal pathogens (122). Nasal vaccines in particular produce respiratory T_RM_ cells more efficiently than injective vaccines (113). There are several possible ways in which exercise training might improve the production of T_RM_ cells elicited by vaccination.

The exercise done during the process of vaccination may help to facilitate the migration of T cells to the vaccination location and draining lymph nodes, where antigens are met and first primed (119). Inflammation caused by exercise might offer danger signals that make vaccines immunogenic. The metabolic fitness of T cells can be improved with regular exercise training enabling them to survive and become long lived memory populations.

The clinical trials investigating the role of exercise in producing positive vaccine responses are encouraging. A meta-analysis showed that physical activity regularly improves influenza vaccine responses, especially in people aged 65 years and over (123). It is unknown if these effects can be observed in the production of T_RM_ cells specifically.

### 6.4 Cancer immunotherapy

T_RM_ cells are important players in the anti-tumor response, and their presence in the tumor correlates with a positive response to immune-checkpoint inhibitors (61, 62). Preclinical models of exercise training have been shown to promote antitumor immunity, which can be influenced by NK cells and CD8^+^ T cells (124, 125). However, most of these studies demonstrate enhanced CD8^+^ T cell infiltration and effector fitness, rather than directly assessing tumor-infiltrating T_RM_ cells. An important question, which remains unanswered, is whether exercise specifically promotes T_RM_ cell accumulation or functionality within the tumor microenvironment.

The potential mechanisms by which exercise can improve T_RM_ cell mediated antitumor immunity are as follows: (1) increasing the trafficking of T cells to tumors by β2-AR signaling; (2) increasing the metabolic fitness of the T cells enabling them to survive in the tumor microenvironment that is unfavorable due to its metabolism; (3) decreasing immuno-suppression of the tumor by the anti-inflammatory effects.

The combination of exercise and immune checkpoint blockade may have synergistic effects. Preclinical studies show that exercise improves the efficacy of PD-1 blockade in several tumor models (126). There are clinical trials underway exploring exercise during immunotherapy.

### 6.5 Unanswered questions and future directions

In spite of advances in the knowledge of both the T_RM_ cell biology and exercise immunology, there are still fundamental questions at the crossroads awaiting answers:

Causality: Is it a fact that exercise directly causes T_RM_ cells to be built or is the observed correlations simply due to confounders? Interventional research and longitudinal measurements of the density and functionality of T_RM_ cells will be necessary to obtain definitive evidence. Mechanistically understanding might be achieved by using animal model systems of T_RM_ cells with reporter systems.

Dose-response: relationship between T_RM_ cell increase and exercises: Dose-response question: When it comes to promoting the growth of T_RM_ cells, which amount of exercise, represents to be optimal? In what way do intensity, frequency and duration work together to define the outcome? This kind of research requires methodologically controlled dose response studies in animal models and then subsequent translational studies in humans.

Tissue specificity: Does exercise have a differing effect on T_RM_ cells in various tissues? There is a possibility that respiratory T_RM_ cells can be selectively improved because of the high pulmonary blood flow during exercise, however, there are no direct comparisons.

Mechanisms: What are the molecular mechanisms that mediate the effects of exercise on T_RM_ cells? The β2-AR signaling pathway is one of the top contenders in this regard, but additional inputs by other pathways (i.e., hemodynamic shear stress, thermal stress, myokines) should be investigated. Genetic and pharmacological loss-of-function experiments could demonstrate causality.

Duration: What is the duration of exercise-induced T_RM_ cell modulations? Must an exerciser keep up a sustained practice, or will there be opportunities at times to provide periodic boosting activities to preserve improved T_RM_ cell immunity?

Aging and sickness: Is it possible that physical activity helps boost immunity of T_RM_ cells in elderly and immunosuppressed people who are the most prone to infectious diseases? The aging process leads to a decrease of T_RM_ cell generation and exercise might possibly inhibit such phenomenon (127).

Gender differences: Are there any differences in T_RM_ cells response between males and females to exercise? The sex hormones affect the differentiation and activity of the T cells and exercise responses could have a sexual dimorphism (128).

## 7 Conclusion

This discovery highlights the fact that T_RM_ plays a crucial role in local immunity at the body surfaces, not the circulating cells, which redefines immunological memory. This discovery has significant implications for exercise immunology, which has been focused on circulating immune cells.

The hypothesis for this exercise immunology is that exercise training can improve the function of T_RM_ cells, including those in non-lymphoid tissues such as the respiratory tract mucosa. The hypothesis also states that adrenergic signals from exercise, together with hemodynamic forces, thermal stress, and metabolic responses, can improve T cell trafficking to tissues, T_RM_ differentiation, and long-term maintenance. This hypothesis explains the reduced risk of upper respiratory infections in exercise-trained persons.

This hypothesis also explains the J-shaped relationship between exercise volume and infection risk, which suggests that moderate exercise, optimizes T_RM_ immunity, but extreme exercise training impairs T_RM_ support. This hypothesis provides new avenues of investigation, such as the role of β2-AR signaling, tissue-specific exercise effects, and exercise effects on vaccination and cancer immunotherapy.

For this hypothesis to be effective, collaboration between immunologists, exercise physiologists, and clinicians is required for its success. Non-invasive immune monitoring will be required for testing this hypothesis in the context of the human immune response, while cell-type specific manipulation models will be required for determining causality.

Ultimately, this hypothesis has significant implications for exercise immunology, which could be shifted from a focus on circulating cells to a spatial immune remodeling approach. This spatial approach represents the future of exercise immunology, which is still in its infancy.
